# Vaccination of domestic animals with a novel oral vaccine prevents *Giardia* infections, alleviates signs of giardiasis and reduces transmission to humans

**DOI:** 10.1038/npjvaccines.2016.18

**Published:** 2016-09-15

**Authors:** Marianela C Serradell, Alicia Saura, Lucia L Rupil, Pablo R Gargantini, Marcela I Faya, Paulina J Furlan, Hugo D Lujan

**Affiliations:** 1 Centro de Investigación y Desarrollo en Inmunología y Enfermedades Infecciosas. Consejo Nacional de Investigaciones Científicas y Técnicas (CONICET), Cordoba, Argentina; 2 Facultad de Medicina, Universidad Católica de Córdoba, Cordoba, Argentina; 3 Facultad de Veterinaria, Universidad Católica de Córdoba, Cordoba, Argentina

## Abstract

*Giardia lamblia* is a human intestinal parasite and one of the most frequent enteric pathogen of companion animals. Clinical manifestations of giardiasis, such as diarrhoea, anorexia, weight loss and lethargy, have been associated with *Giardia* infections in both domestic and farm animals. A few anti-parasitic drugs are routinely used to treat giardiasis, but re-infections are common and drug-resistant strains have already been reported. Unfortunately, efficient vaccines against *Giardia* are not available. *Giardia* undergoes antigenic variation; through this mechanism, parasites can avoid the host’s immune defenses, causing chronic infections and/or re-infections. Antigenic variation is characterised by a continuous switch in the expression of members of a homologous family of genes encoding surface antigens. In a previous report, we indicated that in *Giardia*, the mechanism responsible for the exchange of variant-specific surface proteins (VSPs) involves the RNA interference (RNAi) pathway. From a repertoire of ~200 VSP genes, only one is expressed on the surface of single trophozoites; however, RNAi machinery disruption generates trophozoites that express the complete VSP repertoire. We also demonstrated that gerbils orally immunised with VSPs isolated from these altered parasites showed high levels of protection. Here we tested this vaccine in cats and dogs, and found that it is highly efficient in preventing new infections and reducing chronic giardiasis in domestic animals both in experimental and natural infections. Remarkably, immunisation of dogs in a highly endemic area strongly decreased the percentage of infected children in the community, suggesting that this vaccine would block the zoonotic transmission of the disease.

## Introduction

The flagellated protozoan *Giardia lamblia* (syn. *G. duodenalis* or *G. intestinalis*) is one of the most common causes of intestinal disease in humans and waterborne outbreaks of diarrhoea, as well as a frequent cause of diarrhoea in day-care settings, institutionalised populations and travellers.^1,2^ In regions characterised by deficient basic sanitation, *Giardia* infections in children are almost general.^3^ Although *G. lamblia* is recognised worldwide as the most frequent protozoan parasite causing intestinal disease in humans, the relevance of *Giardia* infections in other mammals is controversial.^4^ Domestic dogs and cats can be asymptomatic or suffer diarrhoea due to maldigestion, malabsorption and augmented gut motility. In companion animals in particular, *Giardia* is regarded as a potential cause of diarrhoea, weight loss and lethargy.^5^

The life cycle of *Giardia* includes the infective cyst and the vegetative trophozoite. Infection is spread to a number of different hosts that ingest cysts, which are eliminated with the faeces.^6^ Once flagellated trophozoites are excysted, they colonise the host’s upper small intestine. Trophozoites are not invasive and proliferate attached to the surface of the intestinal epithelium via a ventral disk.^2^ Trophozoites are the agents responsible for the clinical manifestations of the disease, which may range from asymptomatic infection to acute or chronic diarrhoea. Some individuals are chronically infected and could have no symptoms, but may suffer malabsorption.^7^ The immune status of the host influences its susceptibility to infection as well as the severity of the clinical manifestations. In humans, children and the elderly are especially vulnerable to *Giardia*.^7^ In companion animals, puppies and kittens are more susceptible than adult animals.^8^ In all species, symptoms usually appear 1–2 weeks after infection and persist for several days.^7,9^

Much of veterinary research has studied *Giardia* prevalence and performed molecular characterisation of isolates obtained from different hosts to determine their zoonotic potential.^10^ Eight *G. lamblia* assemblages (A–H) were identified via phylogenetic analyses.^11,12^ Assemblages A and B were found to be capable of infecting several mammals that can become reservoirs of human infections.^12^ Humans can also be a possible *Giardia* reservoir for domestic and production animals.^13^ *G. canis* (assemblage C), *G. cati* (assemblage F) and *G. bovis* (assemblage E) would be limited to certain species or host types,^12^ whereas other *Giardia* species have a broad host range, including the human assemblages A and B.^10–12^ In addition to the livestock assemblage E, the zoonotic assemblage A and, on occasions, the human assemblage B have been found in production animals.^14^

*Giardia* undergoes surface antigenic variation, a mechanism by which parasitic microorganisms can evade the host’s immune response.^9,15^ Antigenic variation in *Giardia* involves variant-specific surface proteins (VSPs). Immune responses to *Giardia* involve a strong humoral immune response to VSPs.^9^ A humoral immune response in experimentally as well as naturally *Giardia*-infected hosts occurs along with the elimination of the original VSP; hence, a functional role of antibodies in selecting phenotypic variants during the course of infection has been suggested.^15^ In addition, monoclonal antibodies (mAbs) of different immunoglobulin subtypes specific to different VSPs were found to be cytotoxic to trophozoites expressing a particular antigen.^16,17^

Of a repertoire of over 200 homologue genes encoded in the parasite genome,^18^ only 1 VSP is expressed on the surface of every single trophozoite at any given moment;^9,15^ however, switching in expression to an antigenically distinct VSP has been reported to occur even in the absence of any immune pressure.^2,9^

Although antigenic variation in *Giardia* has been known for decades, the molecular basis for its regulation remained elusive. In 2008, we reported that, in this parasite, a mechanism similar to RNA interference is involved in the control of the expression of surface antigens.^19^ We found simultaneous transcription of several, or all, VSP genes (*vsp*s); however, a system containing a constitutively expressed RNA-dependent RNA-polymerase (RdRP) produces antisense RNA, targeting all *vsp* transcripts, except one. The duplexes generated between the sense and the antisense *vsp* RNAs are further cleaved to 22–25 nt interfering RNAs by a Dicer/Argonaute system, both *in vitro* and *in vivo*. Notably, evidence obtained from knockdown experiments showed that RdRP and Dicer are involved in the change of expression from a single to multiple VSPs in individual *Giardia* trophozoites.^19^

We have recently hypothesised that trophozoites expressing the complete VSP repertoire on their surface would confer protection against future infections. Therefore, we performed experiments using altered parasites of the *Giardia* WB isolate (assemblage A) in the gerbil model of giardiasis. Our results showed that the animals initially infected with cells expressing all of the VSPs encoded in their genome are largely protected from infection by *Giardia* clones that express a unique VSP on their surface or by cysts obtained from infected individuals.^20^ In addition, immunisation with the entire repertoire of VSPs purified from these transgenic cells, using a mAb directed to the 5-amino-acid conserved cytoplasmic tail, also provided protection against secondary infections.

In this work, we used wild-type and transgenic *Giardia* parasites to determine the course of experimental infections in young and adult dogs and cats. Subsequently, we used an oral vaccine formulation comprising VSPs immunopurified from trophozoites of the *Giardia* WB isolate expressing the full repertoire of their VSPs and tested the vaccine efficacy in experimental infections in dogs inhabiting a *Giardia* endemic shanty town. Remarkably, vaccination of pets in that disadvantaged community decreased the rate of infection in children, demonstrating the zoonotic potential of *Giardia* parasites and the efficacy of this oral vaccine.

## Results

**Figure 1:**
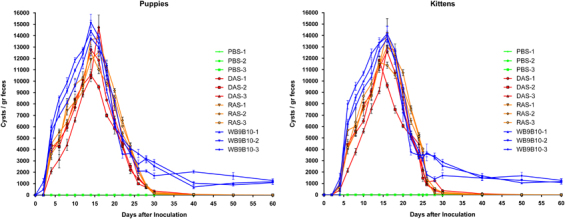
Detection and quantification of *Giardia* cysts in stool samples of domestic animals infected with different populations of wild-type and transgenic trophozoites. Faeces from individually housed puppies (*n*=12) and kittens (*n*=12) were collected, resuspended in PBS, examined with a FITC-labelled mAb 7D6 anti-CWP2 and counted every other day for 2 months. Animals were infected with a clonal population of trophozoites of the WB9B10 isolate (*n*=3) or transgenic trophozoites in which Dicer (DAS; *n*=3) or RdRP (RAS, *n*=3) was knocked down. Three animals received PBS only and served as negative control and as sentinel of environmental conditions. Values represent the mean±s.d. of cyst count performed in triplicate in each animal.

Initially, experimental infections were induced in puppies and kittens to determine whether the results obtained in the gerbil model of giardiasis^20^ are similar to those in domestic animals. We generated trophozoites of the WB isolate expressing the entire repertoire of VSPs by knocking down the expression of *Giardia* Dicer (DAS) or *Giardia* RdRP (RAS).^19^ As shown previously,^20^ these transgenic trophozoites grow and encyst *in vitro* and *in vivo* as wild-type cells. These *Giardia* populations and the wild-type WB clone expressing VSP9B10 were initially used to infect *Giardia*-naive puppies and kittens (a total of 12 kittens and 12 puppies, 3 animals per experimental group). Infection was initiated by orogastric inoculation of trophozoites or control phosphate-buffered saline (PBS). Cyst release, which clearly indicates animal infection and completion of the parasite life cycle, was evaluated by identifying *Giardia* cysts in stool samples via immunofluorescence assays using a mAb specific for *Giardia* cyst wall protein2 (CWP2; mAb 7D6). Figure 1 shows that all populations were able to establish infection in both puppies (left) and kittens (right).

The beginning of cyst detection in stool samples and the number of cysts varied slightly among the different trophozoites and animals used in this study. Infected animals presented some clinical manifestations of giardiasis between the second and fourth weeks of infection (Table 1). However, most of the animals infected with trophozoites that express the entire repertoire of VSPs were able to self-cure by day 40 post infection, whereas the animals infected with a clonal population expressing a unique VSP remained chronically infected, showing recurrent and intermittent clinical manifestations. The animals were treated before challenge experiments at day 60 post infection to avoid possible chronic infection with a small number of trophozoites and to completely eradicate any possible infection.

**Figure 2:**
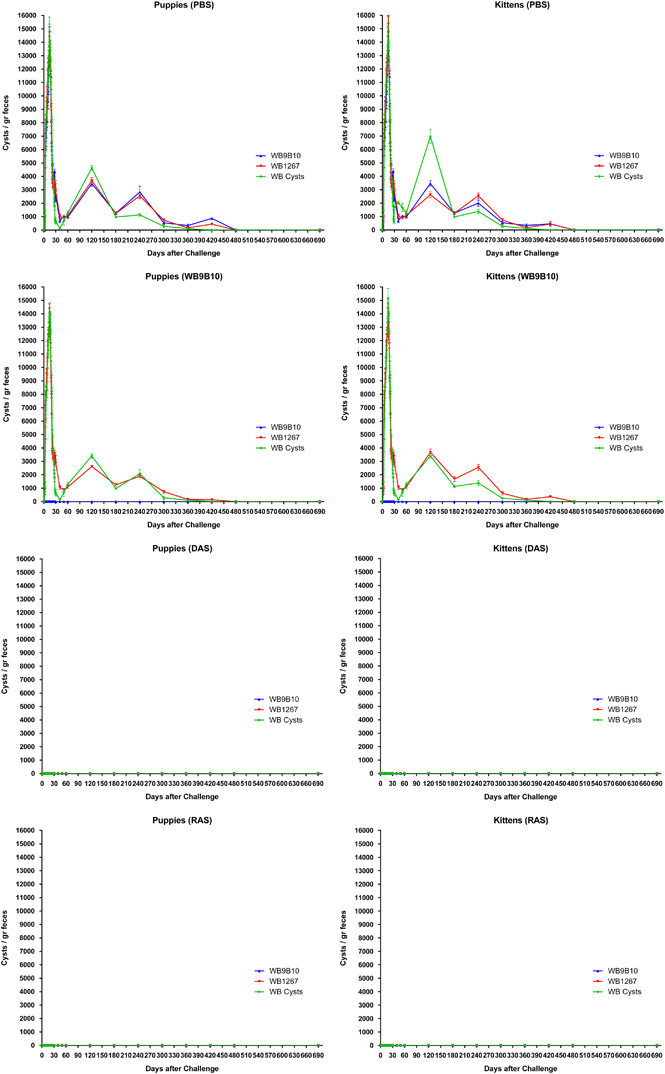
Detection and quantification of *Giardia* cysts in stool samples of domestic animals infected with different populations of wild-type and transgenic trophozoites and challenged with wild-type trophozoites or cysts. Animals infected with a clonal population of trophozoites of the WB9B10 isolate, or DAS or RAS transgenic trophozoites from the experiment shown in Figure 1 were treated with anti-parasitic drugs and then challenged with wild-type trophozoites of clone WB1267, clone WB9B10 or with cysts obtained from infected individuals. Subsequently, faeces were analysed for the presence of cysts by Immuno Fluorescence Assay. Values represent the mean±s.d. of cyst count performed in triplicate in each animal.

To determine whether animals previously infected with trophozoites expressing a particular VSP (VSP9B10) or with cells expressing the entire repertoire of VSPs (DAS and RAS) were protected against subsequent infections, animals previously infected as in Figure 1 were inoculated with clonal populations of trophozoites expressing a specific VSP (VSP9B10 or VSP1267) or with fresh cysts obtained from gerbils, 2 months after the cure of the primary infection. The results of cyst elimination in puppies or kittens (Figure 2) clearly indicated that: (a) control animals (PBS) were readily infected with any *Giardia* parasite; (b) all animals infected with trophozoites expressing a unique VSP were refractory to a secondary infection with cells expressing the same VSP; (c) animals infected with cells expressing a particular VSP were easily re-infected with trophozoites expressing a different VSP; and (d) most animals that were initially infected with trophozoite populations expressing the entire repertoire of VSPs (DAS and RAS) were protected from future infections with clonal populations expressing a particular VSP on their surface or with cysts (Figure 2). The same challenge experiments were performed 4, 12, 18 and 24 months after the original infection, showing the same results (data not shown).

**Figure 3:**
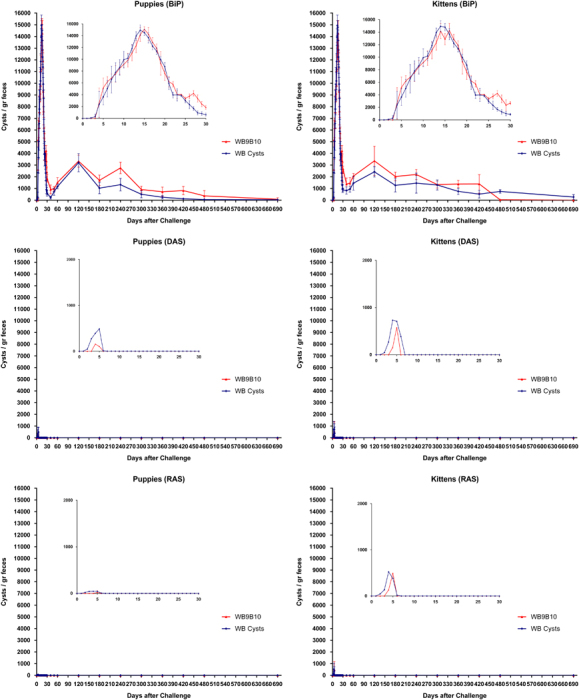
Detection and quantification of *Giardia* cysts in stool samples of domestic animals previously immunised with BiP or VSPs purified from different clonal trophozoite populations and challenged with wild-type trophozoites or cysts. Immunisations were performed as explained in Methods using BiP or VSPs purified from DAS and RAS transgenic trophozoites. After oral vaccination animals were challenged with wild-type trophozoites of clone WB1267, clone WB9B10 or with cysts. Subsequently, faeces from individually housed puppies (*n*=30) and kittens (*n*=30) were collected, resuspended in PBS, labelled with a FITC-labelled mAb 7D6 anti-CWP2 and counted every other day for 2 months. Values represent the mean±s.d. of cyst count of each group (*n*=5). Insets represent a magnification of the first 30 days after challenge.

On the basis of our previous results in gerbils and those observed in puppies and kittens, the entire repertoire of VSPs from the transgenic cells was immunopurified using a mAb that reacts with the 5-amino-acid cytoplasmic tail present in all known VSP molecules.^21^ As a control, an internal, highly immunogenic antigen of *Giardia*, the molecular chaperone of the endoplasmic reticulum BiP,^22^ was immunopurified from *Giardia* homogenates and resuspended in the same buffer. Puppies and kittens were then immunised with these protein preparations without adjuvants by orogastric administration. After 2 months, animals were infected with cysts or with a parasite population expressing particular VSPs (Figure 3). The infection was monitored by counting the cysts released in the stool and by performing an endoscopic study at day 16 post infection to detect the presence of trophozoites in the small intestine. As in the results observed during primary infections with transgenic trophozoites, immunisation with the complete repertoire of VSPs produced an immune response in which cyst shedding ceased very rapidly and the numbers of cysts released were also significantly reduced. By contrast, control animals inoculated with BiP were easily infected by a clonal population of trophozoites (Figure 3). No signs of giardiasis or adverse reactions were observed in the vaccinated animals, indicating that the clinical manifestations of the disease are produced by the entire trophozoites and not by the purified VSPs. Remarkably, unvaccinated dogs showed clinical signs of giardiasis and intermittent shedding of cysts for a long time (Figure 3).

**Figure 4:**
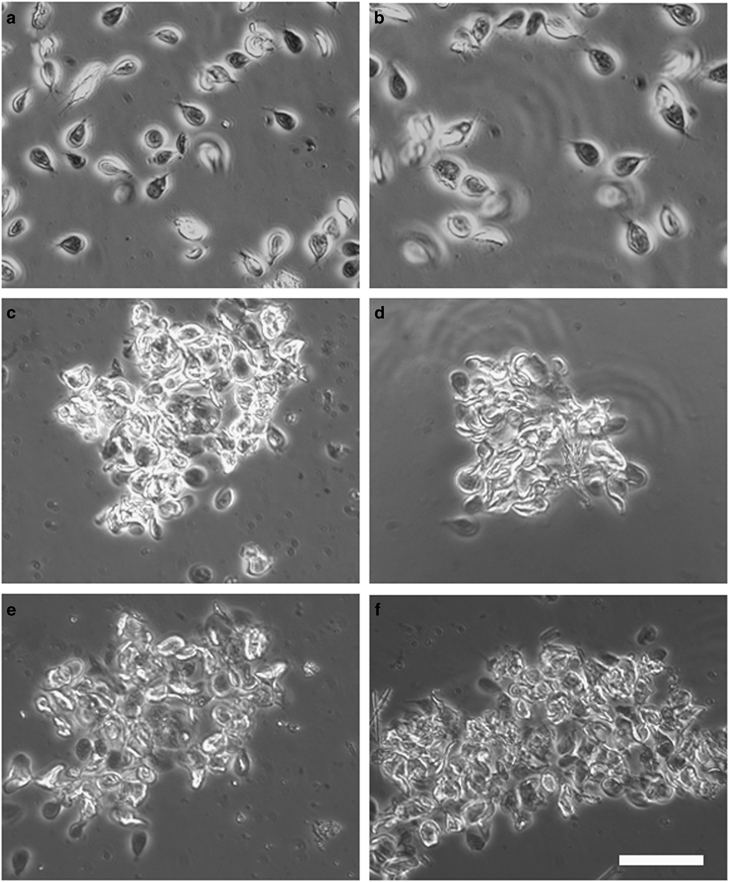
Serum and intestinal content of domestic dogs immunised with the entire repertoire of VSPs can agglutinate different *Giardia* clones *in vitro*. Phase-contrast microscopy of *G. lamblia* clone WB9B10 confronted *in vitro* with serum (**a**,**c**,**e**) and intestinal content (**b**,**d**,**f**) (diluted 10-fold with culture medium) from dogs immunised with either BiP (**a**,**b**) or the entire repertoire of VSPs: DAS (**c**,**d**) or RAS (**e**,**f**). White bar represents 100 μm.

In addition, serum and intestinal content were collected and confronted *in vitro* with trophozoites expressing one or various VSPs. Serum or intestinal content of BiP-immunised animals had no effect on parasite morphology, viability or motility (Figure 4). By contrast, when any clonal population was confronted with serum or intestinal content of animals infected with cells in which Dicer had been silenced (DAS), agglutination of almost all the trophozoites occurred (Figure 4). These results indicate that the infected puppies and kittens were able to develop an effective humoral response to the VSPs present on the trophozoites.

**Figure 5:**
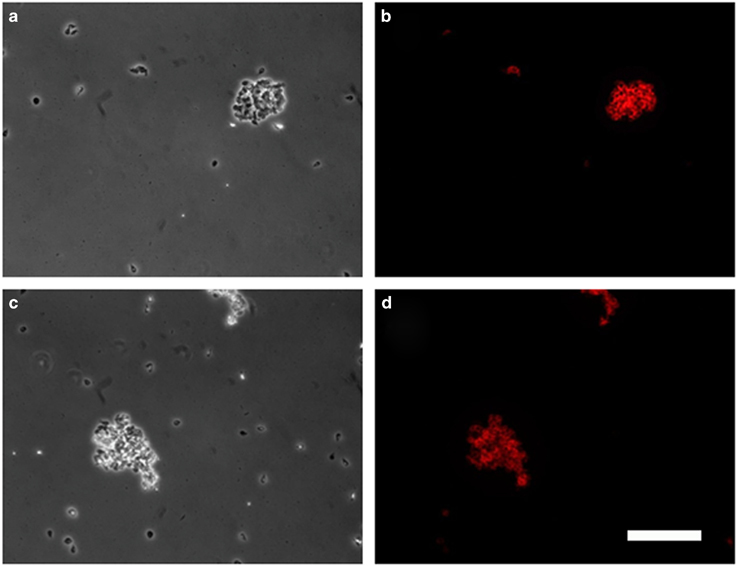
Intestinal content of a dog vaccinated with VSPs purified from DAS-altered trophozoites contains antibodies against VSPs of *G. lamblia* assemblage B and G. muris. Phase-contrast (**a**,**c**) and immunofluorescence microscopy (**b**,**d**) of *G. lamblia* clone GS/M-H7 from assemblage B (**a**,**b**) and *G. muris* obtained from naturally infected experimental rats (**c**,**d**) confronted with intestinal content from a dog immunised with the entire repertoire of VSPs (DAS). An anti-dog IgA (red) was used for immunofluorescence microscopy. White bar represents 100 μm.

We also tested intestinal fluids against trophozoites of the isolate GS/assemblage B and of *G. muris* (Figure 5). In these cases, intestinal content of animals immunised with VSPs purified from the WB isolate/assemblage A showed partial cell agglutination (between 70 and 80%), indicating that *Giardia* from different assemblages, or even species, may share common epitopes in their VSPs. By contrast, in the gerbil model it was not possible to determine immunoglobulin subtypes.^20^ Here we found that all anti-VSPs Ig subtypes were found in the sera of the infected animals, whereas only sIgA could be detected in the intestinal contents of immunised animals, indicating both local and systemic immune responses to *Giardia* VSPs (Table 2).

On the basis of the promising results of the vaccine preparation in experimental studies in gerbils,^20^ and in puppies and kittens (this study), we decided to vaccinate a group of dogs and cats present in a poor, peri-urban community of Córdoba, Argentina. The presence of giardiasis in children living in this shanty town as well as in dogs and cats roaming freely in this area was tested using a simple and rapid immunodiagnostic dipstick device, which has been developed in our laboratory based on mAbs directed to *Giardia* CWP1 and CWP2. In the initial screening attempted to determine the sensitivity and specificity of the assays compared with immunofluorescence assays, 28 dogs and 16 cats of unknown age and pedigree were analysed. Stool samples showed that 100% of the animals were positive for *Giardia* cysts by both techniques (data not shown). Infection of children living in that community was almost universal (99% in samples from 134 children under 12 years of age tested at least twice). Additional genotyping of purified cysts showed that all samples belonged to assemblage A (data not shown). Infected animals were treated with a combination of anti-helmints and anti-protozoan drugs for 3 days (albendazole and metronidazole, 25 mg/kg per day for both drugs). Subsequently, animals were marked, and 20 dogs and 10 cats were vaccinated with two doses of the oral vaccine comprising the whole repertoire of VSPs of the WB isolate (assemblage A), and released back to the community. The remaining animals were used as control. At random times for a period of 2 years, animals were recaptured, taken to the experimental kennel and tested for giardiasis, as described in Methods. Only 12 vaccinated dogs, 5 vaccinated cats, 4 unvaccinated dogs and 2 unvaccinated cats could be followed for the 2-year period, whereas the others were tested occasionally when found. None of the vaccinated dogs and cats was positive for giardiasis, whereas 70–100% of the unvaccinated animals tested positive at different times during this study (data not shown). Infection in children decreased from almost 100–38% 24 months after animal vaccination. Three therapeutically vaccinated and two unvaccinated dogs were taken to the experimental facility and tested for *Giardia* and signs of the disease. Shedding of cysts into the environment and the sporadic signs of infection observed before vaccination was no longer recorded in the vaccinated dogs (Table 3).

**Figure 6:**
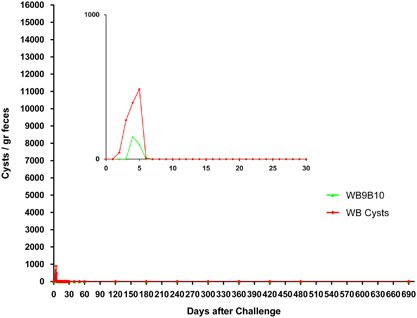
Detection and quantification of *Giardia* cysts in stool samples of puppies previously immunised orally with a plasma membrane preparation of transgenic *Giardia* trophozoites. After vaccination, individually housed puppies (*n*=5 per group) were infected with clonal populations of trophozoites of clone WB9B10 or cysts. Their feces were collected, resuspended in PBS, labelled with a FITC-labelled mAb anti-CWP2 and cysts were counted on different dates. Immunisations were carried out as explained in Methods using a plasma membrane fraction purified from a mixture of DAS and RAS transgenic trophozoites. Values represent the mean±s.d. of cyst count of each group. Insets represent a magnification of the first 30 days after challenge.

The cost of producing our oral vaccine at a large scale includes immunoaffinity purification of the entire repertoire of VSPs from the transgenic parasites. Therefore, this efficient oral vaccine might be considered expensive for veterinary use. For that reason, instead of purifying the entire repertoire of VSPs by immunoaffinity, we used an inexpensive cell fractionation procedure that provides a plasma membrane preparation highly enriched in VSPs. When this fraction was used as an oral vaccine, the obtained results were identical to those observed with immunopurified VSPs, including its high efficiency in preventing the establishment of new infections either by trophozoites expressing particular VSPs or by cysts, and the lack of clinical signs of the disease (Figure 6).

## Discussion

*Giardia* has been reported as a common parasite of dogs and cats worldwide.^23^ The reported prevalence of this parasite in dogs is ~10% in well-cared dogs, 35–50% in puppies and almost universal in breeding facilities and kennels.^24–28^ Prevalence in cats showing signs of gastrointestinal disease has been reported to vary between 6 and 20%, being higher in young animals.^24–28^ To our knowledge, studies in disadvantaged areas lacking basic sanitary conditions have not been reported.

To the best of our knowledge, experimental infections of dogs and cats with human isolates of *Giardia* have been rarely attempted.^13,27^ In this work, we show not only that wild-type parasites can successfully infect domestic animals but also that *Giardia*, whose mechanism of antigenic variation has been disrupted, produces infection and signs of the disease in puppies and kittens, similar to those obtained in the gerbil model of giardiasis.^20^ Animals initially infected with cells expressing the entire repertoire of potential surface antigens were refractory to subsequent infections with trophozoites expressing a unique VSP on their surface or with cysts in which the VSP is unknown. Despite the limited number of animals used, taking together the results from puppies, kittens, dogs and cats, as well as our previous report is gerbils, a reproducible trend is evident.

The extracellular portion of *Giardia* VSPs allows the parasite to overcome the hostile, hydrolytic environment of the upper small intestine. VSPs are then very resistant to variable pH and digestion by intestinal proteases and therefore suitable for oral administration.^20^ In this work, we not only confirmed the general findings obtained in the *Mongolian gerbil* but also demonstrated that vaccine preparation is also effective in larger animals, such as cats and dogs, both prophylactically and therapeutically. During infection with cells expressing the entire repertoire of VSPs cyst shedding was completely avoided when compared with the low number of cysts released in the immunised animals, which may be due to the fact that trophozoites remain longer than the vaccine formulation in the upper portions of the small intestine. These results validate our previous conclusion that antigenic variation is central for the parasite to escape the host’s immune response, and that induction of significant protection against *G. lamblia* requires the entire repertoire of VSPs. Moreover, oral administration of the vaccine preparation to infected dogs, either symptomatic or not for giardiasis, reduced the number of cysts released to the environment and, remarkably, ceased the symptoms of the disease, suggesting that vaccination provides protection from infection as well as parasite clearance post establishment of an infection. Although *Giardia* infections may be symptomatic or asymptomatic in domestic animals, blocking the release of cysts to the environment contributes to a decrease in the rate of infected individuals in hyperendemic areas where basic sanitation practices are deficient.

A commercial vaccine for giardiasis was previously developed for pets and farm animals.^29^ This vaccine consisted of killed trophozoites isolated from a sheep (unknown assemblage) and has been reported to reduce shedding of cysts and prevent clinical disease signs.^30^ The authors showed a decrease or elimination of intestinal trophozoites and faecal cyst excretion in puppies and kittens subcutaneously vaccinated with the trophozoite preparation. Moreover, dogs with signs of chronic giardiasis and that had not responded to chemotherapy were treated with this vaccine preparation. In these cases, vaccination resulted in the cessation of the clinical signs and faecal cyst shedding.^31^ However, results from studies of the efficacy of this vaccine are controversial. A complete study on the efficiency of this vaccine preparation in dogs found no statistically significant differences between the vaccinated animals and the controls at any time regarding shedding of *Giardia* antigens or cysts in stool samples.^32^ This study also determined that this vaccine was not effective for the treatment of asymptomatic canine *Giardia* infections, and that results differed from those previously reported.^32^ These findings are consistent with similar experiments performed in cats^33^ and calves.^34^

Furthermore, studies performed on oral vaccine preparations consisting of recombinant cyst wall proteins^35^ or bacteria expressing CWPs^36^ without the use of adjuvants demonstrated a reduction in the number of cysts released in the faeces of *G. muris*-challenged mice. Those authors propose that these molecules are effective as potential transmission-blocking vaccines. Nevertheless, inhibition of cyst shedding was incomplete either with the recombinant protein or the bacteria expressing CWP, indicating the need for further manipulation of the vaccine preparation. More importantly, our studies showed that cyst shedding was blocked when animals were immunised with the complete repertoire of VSPs, clearly demonstrating that the immune response to these molecules is sufficient to kill the trophozoites before they arrive at the lower parts of the small intestine to begin the process of encystation.^6^ Therefore, our preparations are more effective than the current vaccines not only in protecting the animals against secondary infections but also in avoiding the release of cysts into the environment.

Although we have used a low number of animals because of the long-term isolation needed during the well-controlled challenge experiments, our vaccine preparation using VSPs of a human isolate showed similar results to those observed in the gerbil model of giardiasis, indicating that, again, controlled oral immunisation with VSPs, even in the absence of any adjuvant, can generate a strong immune protection against *Giardia* infections. The antibodies generated by our vaccine comprising VSPs of the WB isolate (assemblage A) showed agglutination of parasites of the GS isolate (assemblage B), and even *G. muris*, clearly indicating that VSPs from different *Giardia* isolates may share common epitopes. Nevertheless, since the efficiency of an anti-*Giardia* vaccine may depend on the VSPs present in different known and still unknown assemblages, it could be necessary to purify VSPs from different isolates that can be cultured in the laboratory to improve the range of our vaccine formulation. Future genomics studies of *Giardia* assemblages different from that used in our studies will allow us to silence the enzymes involved in the regulation of antigenic variation and the generation of transgenic trophozoites expressing the entire repertoire of VSPs from other isolates that can be characterised in the future.

In summary, oral immunisation with the complete repertoire of variable surface antigens hindered establishment of infection by trophozoites or cysts, both in experimental and domestic animals; alleviated disease symptoms in already infected animals; and abolished the release of cysts into the environment, reducing the transmission of the infection to susceptible hosts. These consistent results in three different species (gerbils, dogs and cats) pave the way for the development of a *Giardia* vaccine for humans.

## Materials and methods

### Ethics

All procedures performed on animals were conducted following the protocols specifically approved by the Institutional Committee for Care and Use of Experimental Animals (protocol 2010-36-15p). These protocols adhere to the US PHS (Public Health Service of the United States) guidelines for animal research. Field studies were performed on dogs and cats roving freely in a disadvantaged neighbourhood of Cordoba city, Argentina. Access to those animals was granted by an agreement between the Municipality of Cordoba and the Catholic University of Cordoba (Res. 95/2010). No animals were harmed during the collection of blood, faecal samples or intestinal contents. Faecal samples were taken from children after obtaining written consent of their parents, following the protocols specifically approved by the Bioethics Committee of the Catholic University of Cordoba, which adhere to guidelines of the Ministry of Health of Argentina (Res. 1480/2011).

### Parasites

*Giardia lamblia* WB (ATCC 50803) as well as GS/M (ATCC 50581), clones derived from the WB and GS strains, and transgenic trophozoites were cultured at 37 °C in TYI-S-33 medium in 14-ml borosilicate screw-cap glass tubes.^37^ Trophozoites of *G. muris* were isolated from the small intestine of naturally infected Wistar rats.^38^ *Giardia* clones expressing different surface antigens were obtained by limiting dilution using specific anti-VSP mAbs.^16^ Reactive clones were expanded in culture medium overnight and controlled for homogeneity before use. WB clones 1267 (mAb 5C1), 9B10 (mAb 9B10) and GS/M-H7 (mAb G10/4) were employed in control experiments and challenge infections. Wild-type parasites were genotyped as reported.^39^

### Generation of transgenic trophozoites expressing the whole repertoire of VSPs

Complementary sequences of the genes encoding *Giardia* enzymes RdRP and Dicer were cloned into plasmid pTubHA.pac. Antisense fragments were amplified by PCR from genomic DNA of clone WB/9B10, using oligonucleotide probes containing the *Nco*I and *Eco*RV restriction sites and then cloned into the vector.^19^ *Giardia* was transfected by electroporation and transgenic trophozoites were selected with puromycin. Silencing of these enzymes was verified by quantitative reverse transcription PCR.^19,20^

### Immunofluorescence microscopy

Trophozoites were detached from the tubes by chilling on ice for 20 min. Cells were collected and resuspended in growth medium, and processed for immunofluorescence microscopy, as reported.^19^ A variety of anti-VSP mAbs were used to obtain clonal cell lines expressing a particular VSP and to determine the simultaneous expression of many VSPs in individual trophozoites in a population in which antigenic variation has been deregulated.^19,20^ The generation of mAbs against the CRGKA motif present in all VSPs was described previously.^19^

### Animals

Experimental animals consisted of mixed breed, 6–8-week-old puppies (*n*=52) and 5–8-week-old kittens (*n*=42). The animals were free of *Giardia* as determined by three independent immunofluorescence assays of their faeces using specific mAbs, as well as of any other detectable infectious disease (all animals were completely vaccinated to common bacterial and viral infections before the start of the experimental period: nobivac DP and nobivac HHPPI from MSD Animal Health, Bagovac Rabies from Biogenesis-Bago). Animals were maintained in isolation in individual roofed compartments with the access to an outside run of concrete floor, during the initial infections and during the first 2 months of the challenge experiments. They were offered autoclaved food (commercial dog and cat feed) and sterile water supplemented with a mixture of vitamins (Vigorex, Labyes, Buenos Aires, Argentina) *ad libitum*. All animals were housed under standard operating procedures of the International Animal Care Association. Adult mixed breed dogs and cats from a peri-urban community were tested for the presence of the parasite and treated with anti-parasitic drugs before entering the trial. Each animal was identified with a unique mark. Before infection, all animals were tested for serum antibodies against *Giardia* antigens by enzyme-linked immunosorbent assay using a preparation of total proteins extracted from trophozoites and cysts. The animals having anti-*Giardia* antibodies were not included. Some animals were orally treated with 25 mg of metronidazole and 25 mg of albendazole per kg for 3 days, 10 days before challenges, to rule out any possible presence of low level of *Giardia* in their intestines. Three days before day 0, animals were weighed and randomly allocated to experimental or control groups. The animals were clinically examined every week during the entire trial. Exams involved weight and temperature control, palpation of the abdomen, and evaluation of the appearance, amount and consistency of the faeces.^5^ Following experimental infections, immunisation and challenges of puppies and kittens, faecal samples were collected and analysed for the presence of *Giardia* cysts as described below. Stool samples from adult animals captured from a field community were collected after isolating the dogs and cats under the same conditions as the experimental animals, except that no anti-parasitic treatment were performed before challenges.

### Infections

Infections were induced by orogastric inoculation of 2×10^5^ trophozoites or 1×10^2^ cysts resuspended in 0.5 ml of PBS containing 5 mM cysteine. Control animals were inoculated with 0.5 ml of PBS/cysteine by the same route. In this study, we also used cysts freshly collected from experimentally infected gerbils to prevent samples from rapidly losing viability and infectivity, as previously reported to occur in samples collected from infected individuals.^40^ Faeces from infected animals were collected daily between days 0 and 60 during the initial experiments. Cysts or trophozoites were identified visually via immunofluorescence assays using anti-cyst mAbs^41,42^) or trophozoite-specific antigens (BiP^22^). Excreted *Giardia* cysts were counted by collecting stool samples from individually housed animals over a 24-h period as reported.^20^ The following criteria were used for considering animals uninfected; no cyst was found in the faeces, stool samples were unable to infect experimental gerbils or no trophozoites were detected after 6 days in culture.^43^ For field studies, a *Giardia* cyst-specific immunotest in a dipstick format was developed as described below.

### Production of mAbs to recombinant *Giardia* CWPs and development of a *Giardia* immunotest

mAbs against recombinant CWP1,^41^ CWP2^42^ or protein extracts of freshly isolated cysts derived from the WB isolate were generated as reported.^42^ Hybridomas secreting antibodies were screened by enzyme-linked immunosorbent assay using the original immunogens and by indirect immunofluorescence using encysting trophozoites and cysts. A mAbs anti-CWP1 (mAb 8F12, IgM) was spotted into a nitrocellulose membrane placed on top of a plastic support to capture *Giardia* cysts from stool samples. *In vitro* generated *Giardia* cysts^44^ were used as positive control. The dipstick was then exposed to the anti-CWP2 mAb 7D6 (IgG_2a_) labelled with horseradish peroxidase and subsequently to a color-developing reagent. The minimal amount of cysts that could be detected using this approach was 10 cysts per millilitre of stool samples diluted 50/50 in PBS.

### Purification of VSPs from Dicer-AS and RdRP-AS transgenic trophozoites

Axenic cultures of clones derived from the WB isolate (clones WB9B10 and WB1267), the GS/M isolate (GS/M-H7) and transgenic WB trophozoites expressing the entire repertoire of surface antigens (Dicer-AS and/or RdRP-AS; referred to as DAS and RAS, respectively) were collected as previously reported.^20^ The complete repertoire of VSPs expressed in the transgenic trophozoites were purified by immunoaffinity using the mAb 12F1 generated against the conserved 5-amino-acid tail of VSPs.^19^ Purified VSPs were resuspended in PBS containing 0.01% Tween 20, quantified and used to immunise animals orally.^20^ Protein content in each fraction was quantified using the Bradford’s method. VSP9B10 was purified from a clone expressing only this VSP using the same technique.

### Purification of plasma membrane from transgenic trophozoites

Cultures of the clones expressing the entire repertoire of VSPs were maintained in 14-ml glass tubes fully filled with 5-mm borosilicate glass beads and TYI-S-33 medium containing puromycin. Thus, the total surface where the trophozoites can attach and proliferate was increased approximately four times, whereas the necessary volume of culture medium was reduced by 60%, lowering the cost and highly increasing the number of transgenic trophozoites. These cells were harvested by chilling the tubes for 30 min on ice and the culture medium was collected using Pasteur pipette from the bottom of the tubes. Plasma membranes of sonicated trophozoites were obtained essentially as described.^45^ Fractions of purified membranes containing the full repertoire of VSPs were selected by their reactivity with the mAb 5F12 by western blot, resuspended, quantified and used for oral immunisation.

### Oral immunisations

Animals were immunised with two successive oral administrations of 400 μg of parasite proteins (immunopurified VSPs or isolated plasma membranes of transgenic trophozoites) resuspended in sterile PBS/0.01% Tween 20, 15 days apart. The day of the last administration was considered day 0. Challenges were induced by orogastric inoculation of 2×10^5^ trophozoites or 1×10^2^ cysts resuspended in 0.5 ml of PBS/cysteine. Control animals received 0.5 ml of either PBS/cysteine/Tween or 400 μg of purified *Giardia* BiP in PBS/cysteine/Tween by the same route. Challenges were also performed using cysts freshly collected from experimentally infected gerbils. Faecal samples from immunised animals were taken daily between days 0 and 60, and weekly up to day 600. Cysts or trophozoites were identified visually by immunofluorescence assays. At some time points, selected dogs were subjected to a duodenal endoscopy to collect intestinal fluids, which were added to culture medium at 4 °C for 30 min. The supernatants were collected and examined for *Giardia* trophozoites using light and immunofluorescence microscopy or placed in culture medium for up to 6 days, as reported.^20,43^

### Blood samples

Blood samples were taken after the first day of infection or immunisation to detect the presence of circulating anti-*Giardia* antibodies. Blood was centrifuged at 800*g* for 15 min to obtain serum, which was stored at −70 °C until use.

### Intestinal contents

At day 16 post infection, one animal per group was subjected to an endoscopy.^5^ Animals were previously fasted for 1 day and allowed free access to water. Small intestine fluids were aspirated and collected separately. In some cases, intestinal contents were centrifuged at 5,000*g* at 4 °C to separate cells, debris and bacteria. The supernatants were filter-sterilized and maintained at −70 °C until use in agglutination and immunofluorescence assays.

### Agglutination assays

Assays were performed in 96-well plates with flat-bottom wells. Approximately 5×10^5^ trophozoites per well were incubated at 4 °C for 1 h with various dilutions of intestinal secretions, sera and ascitic fluid containing VSP-specific mAbs (all heat-inactivated) in TYI-S-33 medium without adult bovine serum. The content of wells was mixed by repeated pipetting to assess agglutination of trophozoites under microscope and reveal antibodies using anti-dog or anti-cat immunoglobulins (Abdserotec).

## Figures and Tables

**Table 1:** Clinical signs of giardiasis in experimentally infected puppies and kittens

*Animal/infection*	*Signs*	*Animal/infection*	*Signs*
Puppy 1/PBS	=(0–8)	Kitten 1/PBS	=(0–60)
Puppy 2/PBS	=(0–8)	Kitten 2/PBS	=(0–60)
Puppy 3/PBS	=(0–60)	Kitten 3/PBS	=(0–60)
Puppy 4/WB9B10	Θ ϕ θ (2–5)	Kitten 4/WB9B10	Θ ϕ θ (1–4, 7–8)
Puppy 5/WB9B10	Θ ‡ θ (2–4)	Kitten 5/WB9B10	Θ ‡ (2–5, 8)
Puppy 6/WB9B10	Θ ϕ (2–4)	Kitten 6/WB9B10	Θ ϕ (2–4)
Puppy 7/DAS	Θ ϕ ‡ θ (2–6)	Kitten 7/DAS	Θ ϕ ‡ θ (1–4)
Puppy 8/DAS	Θ ϕ ‡ θ (1–4)	Kitten 8/DAS	Θ ϕ ‡ θ (2–5)
Puppy 9/DAS	Θ ‡ θ (2–4)	Kitten 9/DAS	Θ ‡ θ (2–4)
Puppy 10/RAS	Θ ϕ (3–4)	Kitten 10/RAS	Θ ϕ (1–4)
Puppy 11/RAS	Θ ϕ ‡ θ (2–4)	Kitten 11/RAS	Θ ϕ ‡ θ (2–4)
Puppy 12/RAS	Θ ϕ (3–4)	Kitten 12/RAS	Θ ϕ (2–4)

*Giardia*-infected domestic animals from the experiment shown in Figure 1 exhibited different signs of giardiasis. The following clinical manifestations were recorded: diarrhoea (Θ), weight loss (θ), anorexia (ϕ), lethargy (‡), high temperature (■), allergies (●) or no symptom (=). The week after infection in which the episode occurred is shown between parentheses. No increase in body temperature or allergies was detected in any animal.

**Table 2:** Immunoglobulin subtypes anti-*Giardia* VSPs in infected puppies and kittens

*Animal/infection*	*Serum Ig*	*Intestinal Ig*	*Animal/infection*	*Serum Ig*	*Intestinal Ig*
Puppy 3/PBS	None	None	Kitten 3/PBS	None	None
Puppy 6/WB9B10	A, G, M	A	Kitten 6/WB9B10	A, G, M	A
Puppy 9/DAS	A, G, M	A	Kitten 9/DAS	A, G, M	A
Puppy 12/RAS	A, G, M	A	Kitten 12/RAS	A, G, M	A

Sera or intestinal contents of infected or control animals were used in agglutination assays in 96-well plates. Approximately 5×10^5^ trophozoites were incubated at 4 °C for 1 h with heat-inactivated intestinal secretions and sera in TYI-S-33 medium without bovine serum. The well content was mixed to assess agglutination of trophozoites by microscopy and to reveal antibodies using different anti-dog or anti-cat immunoglobulins.

**Table 3:** Clinical signs of giardiasis in naturally infected dogs after vaccination

*Animal/immunization*	*Week after vaccination*																
	*0*	*1*	*2*	*3*	*4*	*5*	*6*	*7*	*8*	*9*	*10*	*11*	*12*	*13*	*14*	*15*	*16*
Dog 1/PBS	ϕ	Θ ϕ θ	Θ ϕ ‡ θ	‡	=	=	=	‡	Θ ϕ ‡	Θ ϕ ‡ θ	=	=	=	=	‡	Θ ϕ ‡	Θ ϕ ‡ θ
Dog 2/PBS	Θ ϕ ‡	ϕ ‡ θ	Θ ϕ ‡ θ	‡	=	=	=	‡ ϕ	Θ ϕ ‡	Θ ϕ ‡ θ	Θ ϕ ‡ θ	=	=	=	‡	Θ ϕ ‡	Θ ϕ ‡ θ
Dog 3/DAS	ϕ	Θ ϕ θ	Θ ϕ θ	=	=	=	=	=	=	=	=	=	=	=	=	=	=
Dog 4/DAS	Θ ‡	Θ ϕ θ	Θ ‡ ϕ θ	=	=	=	=	=	=	=	=	=	=	=	=	=	=
Dog 5/DAS	Θ ϕ	Θ ϕ θ	Θ ϕ θ	=	=	=	=	=	=	=	=	=	=	=	=	=	=

*Giardia*-infected domestic dogs from the field experiment were vaccinated with the entire repertoire of VSPs purified from DAS trophozoites or control PBS. The following clinical manifestations were analysed over a 4-month period: diarrhoea (Θ), weight loss (θ), anorexia (ϕ), lethargy (‡), high temperature (■), allergies (●) or no symptom (=).
